# Short-term adaptations following Complex Training in team-sports: A meta-analysis

**DOI:** 10.1371/journal.pone.0180223

**Published:** 2017-06-29

**Authors:** Tomás T. Freitas, Alejandro Martinez-Rodriguez, Julio Calleja-González, Pedro E. Alcaraz

**Affiliations:** 1 UCAM Research Center for High Performance Sport, Catholic University of Murcia, Murcia, Spain; 2 Department of Analytical Chemistry, Nutrition and Food Science, University of Alicante, Alicante, Spain; 3 Laboratory of the Sport Performance Analysis, University of Basque Country, Vitoria, Spain; 4 Faculty of Sport Sciences, UCAM, Catholic University of Murcia, Murcia, Spain; Universidade de Tras-os-Montes e Alto Douro, PORTUGAL

## Abstract

**Objective:**

The purpose of this meta-analysis was to study the short-term adaptations on sprint and vertical jump (VJ) performance following Complex Training (CT) in team-sports. CT is a resistance training method aimed at developing both strength and power, which has a direct effect on sprint and VJ. It consists on alternating heavy resistance training exercises with plyometric/power ones, set for set, on the same workout.

**Methods:**

A search of electronic databases up to July 2016 (PubMed-MEDLINE, SPORTDiscus, Web of Knowledge) was conducted. Inclusion criteria: 1) at least one CT intervention group; 2) training protocols ≥4-wks; 3) sample of team-sport players; 4) sprint or VJ as an outcome variable. Effect sizes (ES) of each intervention were calculated and subgroup analyses were performed.

**Results:**

A total of 9 studies (13 CT groups) met the inclusion criteria. Medium effect sizes (ES) (ES = 0.73) were obtained for pre-post improvements in sprint, and small (ES = 0.41) in VJ, following CT. Experimental-groups presented better post-intervention sprint (ES = 1.01) and VJ (ES = 0.63) performance than control-groups.

**Sprint:**

large ESs were exhibited in younger athletes (<20 years old; ES = 1.13); longer CT interventions (≥6 weeks; ES = 0.95); conditioning activities with intensities ≤85% 1RM (ES = 0.96) and protocols with frequencies of <3 sessions/week (ES = 0.84). Medium ESs were obtained in Division I players (ES = 0.76); training programs >12 total sessions (ES = 0.74).

**VJ:**

Large ESs in programs with >12 total sessions (ES = 0.81). Medium ESs obtained for under-Division I individuals (ES = 0.56); protocols with intracomplex rest intervals ≥2 min (ES = 0.55); conditioning activities with intensities ≤85% 1RM (ES = 0.64); basketball/volleyball players (ES = 0.55). Small ESs were found for younger athletes (ES = 0.42); interventions ≥6 weeks (ES = 0.45).

**Conclusions:**

CT interventions have positive medium effects on sprint performance and small effects on VJ in team-sport athletes. This training method is a suitable option to include in the season planning.

## Introduction

In team-sports, the capacity to maximize neuromuscular power production is fundamental to success and critical to achieve high levels of performance and greater velocities in sport specific movements [1]. The improvement of high intensity, explosive actions such as sprint or vertical jump (VJ) is an important goal for coaches and athletes [2, 3]. In fact, Faude et al. [4] concluded that straight sprints are the most important action when scoring or assisting a goal in elite football. For the purpose of this meta-analysis, it is important to state that in most team-sports the distances covered in sprint efforts are usually short [5–7] and consist primarily on accelerations and decelerations without developing full speed [8].

Studies conducted with American Football athletes have shown that Division I players are stronger, faster and more powerful than their Division II or Division III counterparts [9]. Also, Cometti et al. [10] reported that elite soccer players displayed higher strength values and 10 m sprint performance when compared to amateurs. This indicates that strength and power production may differentiate athletes from different competition levels. Therefore, due to the association between these variables and higher performance levels in team-sports, investigating about training methods designed to improve strength and neuromuscular power is of great interest.

Research has shown that resistance training performed with heavy loads as well as programs using light or optimal loads, plyometric training and ballistic exercises lead to increments in maximal power outputs [1], VJ [3, 11–13] and sprint performance [2, 3, 13, 14]. Traditional heavy resistance strength training results in increments in maximal strength and power by targeting mainly the force component of the power equation (power = force x velocity) [1, 15]. However, this type of loading does not play a relevant role in maximal power improvements after reasonable levels of strength are attained [1, 15]. On the contrary, plyometric and ballistic/power exercises performed with lighter loads allow for higher movement velocities to be achieved, which elicits specific adaptations in neural drive that ultimately lead to an increased rate of force development and maximal power production [1, 13, 15]. Finally, methods that combine both strength and power exercises may produce superior improvements in sprint and VJ when compared to strength, power or speed training alone in untrained subjects [12, 16] and athletes [17].

Most recently, Complex Training (CT) has emerged as a training method aimed at developing strength and neuromuscular power. It consists on coupling biomechanically similar heavy load resistance exercises (also referred to as conditioning activities (CA)) with plyometric or power exercises (maximal movement velocities), set for set, in the same workout [18, 19]. Two consecutive exercises combined are termed a complex pair [20] (a back squat followed by a countermovement jump, for example). According to Ebben [19], heavy resistance training increases motoneuron excitability and reflex potentiation, thus possibly creating optimal training conditions for subsequent neuromuscular power gains. Furthermore, Cormie et al. [1] state that the ability to generate maximal power depends greatly on the ability of the nervous system to activate the muscles involved with the adequate order and magnitude of activation.

Theoretically, CT improves performance due to the enhancement of the muscle’s explosive capability after being subjected to maximal or near maximal contractions, in a response known as postactivation potentiation (PAP) [20–22]. The phosphorylation of myosin regulatory light chain [21] and the recruitment of higher order motor units that occurs after maximal muscle contractile activity [21, 23] are the mechanisms believed to contribute to PAP. Seitz and Haff [22] performed a meta-analysis on the factors modulating PAP of jump, sprint, throw and upper-body ballistic performances. According to the authors, performing a CA produces small PAP on jump and moderate on sprint. Furthermore, PAP effects seem to be higher in stronger individuals (squat:body mass ratio ≥1.75 for men and >1.5 for women) and when the CA consists on plyometric drills or resistance exercises ≥85% of 1RM. The results also indicated that the greatest PAP response is obtained after longer recovery intervals (≥5 min) between the CA and the subsequent exercise and also when multiple sets are performed instead of a single one [22]. However, it has also been suggested that CA may have a warm-up effect rather than an actual potentiating one [24] and that this should not be excluded as a possible cause for the improved performance in the subsequent exercise.

CT is considered a time efficient method [25], but there is no clear agreement on its actual effectiveness [26]. Several studies [27–29] investigated its acute effects, mainly focusing on identifying if PAP was present after the CA and if performance increased. Results found were somehow contradicting, since some investigations [29, 30] indicated that CT resulted in subsequent acute increments in power production whereas other studies reported no significantly higher performance gains [27, 28]. Factors like training background [26, 29], subjects’ strength level [20, 29, 31], intracomplex rest interval [22, 26, 31] or the load used in the CA [22, 30, 31] have been proposed as influential in the acute response to CT.

Concerning short- and long-term adaptations, few studies have been conducted to assess the efficacy of CT protocols. Research on recreationally trained individuals indicated that CT did not result in higher whole- and lower-body power output increments when compared to compound training (strength and power sessions on alternate days) [32] or when compared to resistance training only or plyometric training alone [25]. Furthermore, maximal strength adaptations were similar in all different training conditions [25, 32]. Regarding team-sports athletes, disparities can be found within the results published in the literature. Faude et al. [33] found increases in lower body maximal strength and VJ height following a CT intervention with soccer players, but no improvements in 10 and 30 m sprint or agility. McMaster et al. [34] reported increases in both maximal strength and sprint ability in rugby players following CT and Alves et al. [35] obtained significant improvements in sprint (5 and 15 m) but not in countermovement jump or agility performance in soccer players. Other studies reported increases in sprint [36, 37] or VJ [38–40] performance or no positive adaptations on these variables after a several weeks CT program [41].

It remains controversial as to whether CT has a positive effect on sprint or VJ in team-sports but a recent meta-analytical review on the effects of resistance training in youth athletes concluded that for muscular power development, CT provided a greater magnitude of change compared with other resistance training protocols [42]. This suggests that CT may be a promising method to develop neuromuscular power and athletic performance but further understanding on how to organize its training variables is necessary.

Therefore, the main aim of this meta-analysis was to examine the effects of short-term CT interventions (at least 4 weeks) on sprint and VJ performance in team-sport athletes and to identify the possible moderating factors contributing to such adaptations.

## Methods

### Literature research and data sources

This research was completed in accordance with the recommendations of the PRISMA statement (S1 Table) [43]. The literature research was conducted in different online databases: PubMed MEDLINE, SPORTDiscus and Web of Knowledge (WoS). The search included studies published until July 2016 and the following keywords were introduced, either individually or combined: “complex training”, “postactivation potentiation”, “performance”, “athletes”, “players”, “sprint” and “jump”. Reference lists from relevant articles were also scrutinized to find other potentially eligible studies.

### Inclusion and exclusion criteria

Crossover, randomized, non-randomized and counterbalanced studies published in English were considered for inclusion and no age or sex restrictions were imposed. Studies were included if the following criteria was met: 1) at least one of the study’s group was submitted to a CT intervention containing lower-body exercises, in which CT consisted of biomechanically similar (same movement pattern) heavy load resistance training exercises combined with plyometric/explosive exercises, set for set, in the same workout [18, 19]. Studies that combined strength training and plyometric in a different manner (e.g. all strength exercises in the first part of the workout and all plyometric in the end of the session) were not considered; 2) interventions were of at least 4-weeks; 3) participants were athletes currently engaged in team-sport activities, and presented no cardiovascular, metabolic, or musculoskeletal disorders and no history of doping or drug abuse; 4) sprint or VJ were outcome variables measured.

With respect to the exclusion criteria, studies were not considered if: 1) the article was not published in English; 2) no full-text was available; 3) no CT intervention group was present; 4) only acute effects were investigated; 5) participants were not team-sport athletes; 6) sprint or VJ were not outcome variables;

### Study selection

The initial search was conducted by one researcher (TTF). After the removal of duplicates, titles and abstracts were screened and studies not related to the review’s topic were excluded. Following the first screening process, the full version of the remaining articles was read. Then, on a blind, independent fashion, two reviewers selected the studies for inclusion (TTF and AMR), according to the criteria previously established. If no agreement was obtained, a third party intervened and settled the dispute.

### Data extraction and analysis

Mean, standard deviation (SD) and sample size data were extracted by one author (TTF) from tables of all included papers. Whenever necessary, contact was made with the authors to get the data. Any disagreement was resolved by consensus (TTF, AMR), or third-party adjudication (PEA). The meta-analysis and statistical analyses were performed using Review Manager software (RevMan 5.2; Cochrane Collaboration, Oxford, UK) and Comprehensive Meta-analysis software (Version 2; Biostat, Englewood, NJ, USA). For each study, mean differences and 95% confidence intervals (CI) were calculated with Hedges’ g [44] for continuous outcomes.

Each mean difference was weighted according to the inverse variance method [45]. Since sprint time and VJ height were assessed by different methods, the mean differences were standardized by dividing the values with their corresponding SD. The standardized mean difference (SMD) in each trial was pooled with a random effects model [46]. The ESs were calculated using Cohen’s d with the following equation [47], for paired samples:

$$\text{ES}=\frac{{\text{M}}_{\text{pre}}\;\text{-}\;{\text{M}}_{\text{post}}}{{\text{SD}}_{\text{pre}}}\left(1\;\text{-}\;\frac{3}{4n\;\text{-}\;5}\right)$$

where M_pre_ is the mean value before the CT intervention, M_post_ is the mean after the intervention, *n* is the sample size of CT group and Spre is the SD pre-intervention. Additionally, for independent samples (training and control groups (CG)), the ESs were calculated with the formula [47]:

$$\text{ES}=\frac{{\text{M}}_{1}\;\text{-}\;{\text{M}}_{2}}{{\text{SD}}_{\text{pooled}}}$$

where M_1_ is the mean value of the intervention group post CT intervention, M_2_ is the mean of the CG after the intervention and the SD_pooled_ is calculated:

$${\text{SD}}_{\text{pooled}}=\sqrt{\frac{{{\text{SD}}_{1}}^{2}+{{\text{SD}}_{2}}^{2}}{{\text{SD}}_{\text{pooled}}}}$$

ESs were considered small (ES = 0.2), medium (ES = 0.50) and large (ES = 0.80) [47]. The data analysis was focused on the magnitude of effects obtained.

Heterogeneity among studies was assessed using I^2^ statistics. I values range between 0% and 100% and are considered low, modest or high for <25%, 25±50%, and >50%, respectively. Heterogeneity may be assumed when the p-value of the I test is <0.05 and may be due to the variability between the characteristics of the participants of the studies included (age, sex, etc) [48].

Potential moderating factors were evaluated by subgroup analysis comparing trials grouped by dichotomous or continuous variables potentially influencing sprint time and VJ height. Median values of continuous variables were used as cut-off values for grouping trials. Changes in potential moderating factors were expressed and analysed as post minus pre-intervention values. Publication bias was evaluated by estimating funnel plot asymmetry test. Statistical significance was considered for p≤0.05.

### Risk of Bias

Methodological quality and risk of bias were assessed by visual interpretation of the funnel plot, by two authors independently (TTF, AMR), with disagreements being resolved by third part evaluation (PEA), in accordance with the Cochrane Collaboration Guidelines [45].

## Results

### Characteristics of included studies

**Fig 1:**
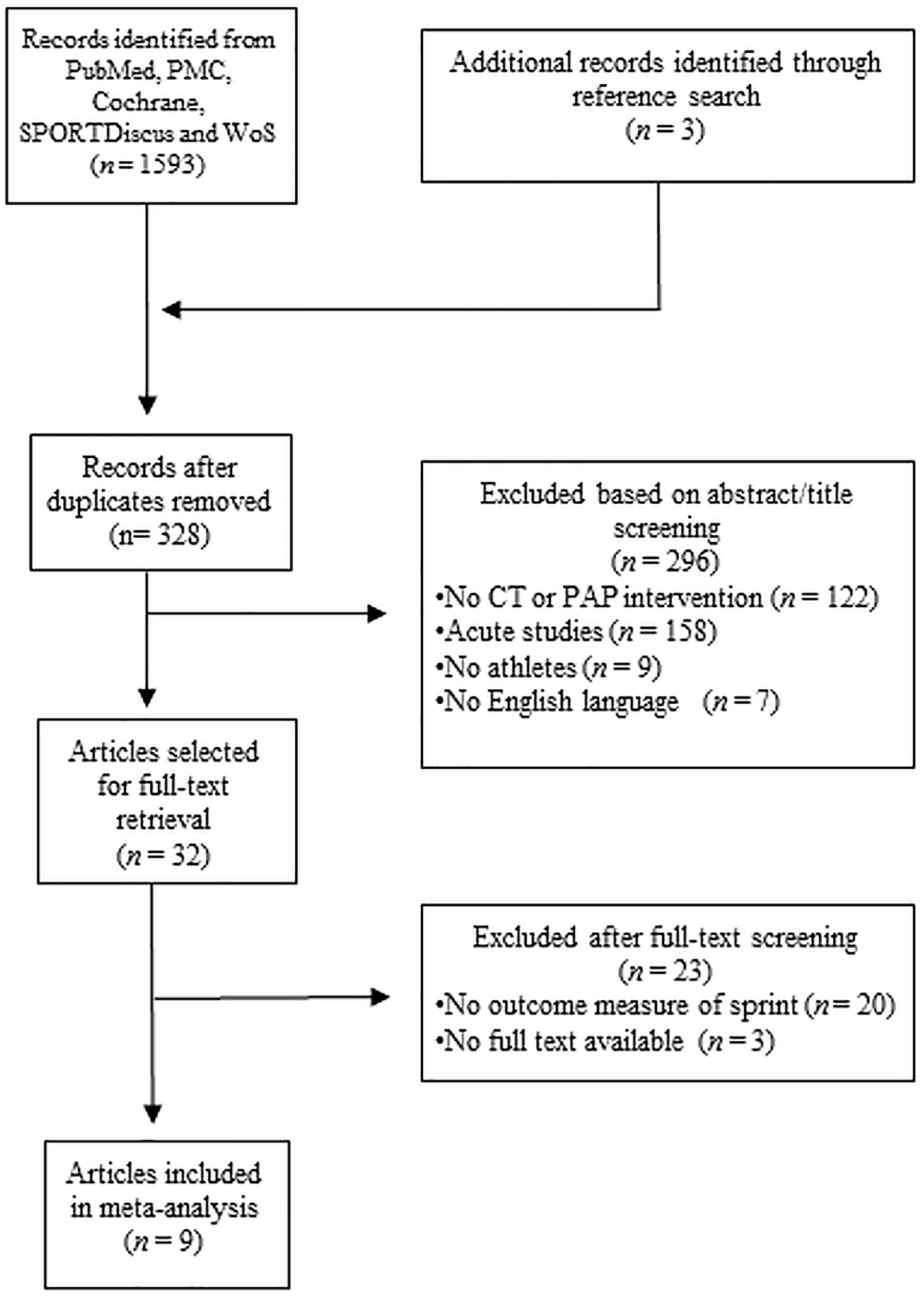
Flow diagram of the process of study selection.

A total of 1593 records were identified through database searches and 3 studies through reference lists. After abstract screening, from the 328 studies that were left following duplicates removal, 296 studies were excluded. As a result, 32 studies were assessed for eligibility. Of these, 23 were excluded for not meeting the inclusion criteria. Consequently, 9 studies [33–41] were included in this meta-analysis (Fig 1).

From the studies included, 4 [34, 35, 38, 41] presented two CT groups which accounted for a total of 9 subgroups analysed for the sprint variable and 8 for VJ. A CG was present in 5 of the studies [33, 35, 37, 40, 41].

The quality of the trials, according to a PEDro scale [49] was high. The mean score was 6.44 ± 1.01 out of a possible 10 points (Table 1).

**Table 1:** PEDro scale scores of the studies included in the meta-analysis.

PEDro Scale Items	Alves et al., 2010	Brito et al., 2014	Cavaco et al., 2014	Dodd et al., 2007	Faude et al., 2013	Kukric et al., 2012	McMaster et al., 2014	Mihalik et al., 2008	Watts et al., 2014
**1. Eligibility criteria (item does not score)**	1	1	1	1	1	1	1	1	1
**2. Random allocation**	1	1	1	1	1	1	1	-	1
**3. Concealed allocation**	1	1	1	1	1	1	1	-	1
**4. Similar groups at baseline**	1	1	1	1	1	-	1	-	1
**5. Blinding of subjects**	-	-	-	-	-	-	-	-	-
**6. Blinding of therapists**	-	-	-	-	-	-	-	-	-
**7. Blinding of assessors**	-	-	-	-	-	-	-	-	-
**8. Measure of one key outcome– 85% of subjects**	1	1	1	1	-	1	1	1	1
**9. Intention to treat**	1	1	1	1	1	1	1	1	1
**10. Between-group comparison**	1	1	1	1	1	1	1	1	1
**11. Point estimates and variability**	1	1	1	1	1	1	1	1	1
**Total Score**	7	7	7	7	6	6	7	4	7

### Characteristics of the interventions

The different CT intervention groups’ characteristics are present in Table 2. The intensity of the lower-body heavy resistance exercises performed ranged from 50% to 100% 1RM and the plyometric/power exercises from body mass to 75% 1RM (loaded CMJ). The interventions ranged from 4 to 10 weeks of duration with a frequency of 1 to 4 sessions/week. The distances covered in sprint assessment ranged from 15 to 30 m. Regarding VJ, three studies used a force platform to record jump performance [33, 38, 40], two utilized a Vertec device [36, 39] and one used a jump mat [35].

**Table 2:** Characteristics of the studies included in the meta-analysis and complex training interventions, sprint time and vertical jump assessment.

	n						Complex Training intervention					Sprint			Vertical Jump		
Study, year of publication	CG	CT	♀ (%)	Age	Sport	Level	Type	Freq (wk^-1^)	ICRI	Duration(wks)	Intensity CA	Measure	Units	Distance(m)	Measure	Units	Type
Alves *et al.[35],* 2010	6	9	0	17.4 ± 0.6	Soccer	D1	**CT1:**	1	No data	6	85% 1RM	Photoelectric cells	sec	15	Jump mat	cm	CMJ
							CTP1- Squat + High Skipping										
							CTP2- Calf Raises +VJ										
							CTP3- Leg ext + VJ from seated position										
		8	0				**CT2**:	2									
							Same as CT1										
Brito *et al. [37],* 2014	21	12	0	19.9± 0.5	Soccer	U-D1	**CT**:	2	20 sec	9	85% 1RM	Photoelectric cells	sec	20	N/A	N/A	N/A
							CTP1- Squat + High Skipping										
							CTP2- Calf Raises +VJ										
							CTP3- Leg ext + VJ from seated position										
Cavaco *et al. [41],* 2014	6	5	0	13.8 ± 0.45	Soccer	U-D1	**CT1**:	1	No data	6	85% 1RM	Photoelectric cells	sec	15	N/A	N/A	N/A
							CTP1- Squat + Sprint										
							CTP2 –Squat + Sprint with ball										
							**CT2**:	2									
							Same as CT1										
Dodd *et al. [36]*, 2007	―	32	0	20.5 ± 2.5	Baseball	U-D1	**CT**: 3 HR combined with 3 PLY	2	<10 sec	4	85% 1RM	Hand-held stop watch	sec	18.3	Vertec device	inch	Aba
Faude *et al.[33],* 2013	8	8	0	23.1 ± 2.7	Soccer	U-D1	**CT**: 2 day routine	2	<10 sec	7	90%/ 50–60% 1 RM	Photoelectric cells	sec	30	Force platform	cm	CMJ
							D1: Unilateral Half-Squat + Single Leg Jumps										
							D2: 2 of 4 CTP + 1 soccer- specific activity										
							CTP1- Half-Squat + DJ										
							CTP2- Calf Raises + High Straight Jumps										
							CTP3- Lateral Half-Squat + Lateral Jumps										
							CTP4- Step-ups + Bounding Jumps										
Kukric *et al. [40]*, 2012	10	10	0	16.5 ± 0.5	Basketball	U-D1	**CT**:	2	5 min	10	80% RM	N/A	N/A	N/A	Force platform	cm	Aba
							CTP1- Standing on toes + Single leg jumps										
							CTP2- Leg Press + Jump over Hurdles										
							CTP3- Step forward + Telemark jumps										
							CTP4- Half-Squat + Jump over Hurdles										
McMaster *et al. [34]*, 2014	―	14	0	20.9 ± 1.6	Rugby	D1	**SHB**: 4 day routine	4	2 min	5	85-100/60-75% RM	Photoelectric cells	sec	20	N/A	N/A	N/A
							D1: CMJ + CM bench throws Power Cleans + Jammer Press DB Snatch										
							D2: CTP1- Bench Press + Bench Press Throws										
							CTP2- Chin-ups + High Pulls										
							CTP3- DB Floor Press + DB rows										
							D3: CTP1- Back Squat + Squat Jumps										
							CTP2- Bulgarian Split Squat + Speed Lunge										
							D4: CTP1- Incline DB press+ Alternate DB bench press										
							CTP2- Hip Thrusts + Calf Raises										
							**SLB**: Same as SHB										
Mihalik *et al. [39]*, 2008	―	15	67	20.3 ± 2.2	Volleyball	D1	**CT**: Squat + Depth Jump Single Leg Lunge + Split Squat Jump Deadlift + Double Leg Bounds	4	2 min	4	60% 1RM	N/A	N/A	N/A	Vertec device	cm	Aba
Watts *et al.[38]*, 2014	―	4	0	16.8 ± 0.6	Volleyball	D1	**HRS**: 2 day routine	4	2 min	4	90% 3RM/ 90% 1RM	N/A	N/A	N/A	Force platform	cm	CMJ
							D1: CTP1- Power Snatch + Medicine Ball Throw										
							CTP2- Back Squat + Depth Jumps Front Squat										
							D2: CTP1- Power Clean + Spike Jump										
							CTP2- Front Squat + Standing Long Jumps Deadlift										
		5	0	17.9 ± 1.1			**LRS**: Same as HRS										

Data are mean, mean ± SD, n or range. C = control group; CT = complex training exercise-group; ICRI = Intracomplex Rest Interval; CA = Conditioning Activity; RM = Repetition Maximum; D1 = Division 1; U-D1 = Under Division 1; CTP = Complex Pair; HR = Heavy Resistance Exercises; PLY = Plyometric Exercises; CT1 = Complex Training group I; CT2 = Complex Training group 2; SHB = Strength Heavy Ballistic complex training group; SLB = Strength Light Ballistic complex training group; HRS = High Reactive Strength group; LRS = Low Reactive Strength group

### Main effects analysis

**Fig 2:**
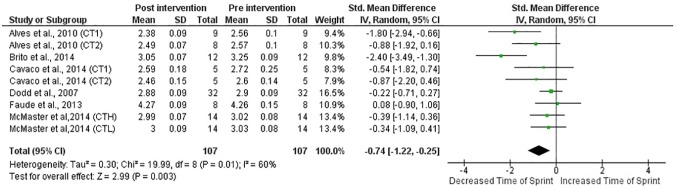
Standardized mean difference (SMD) between post and pre-intervention sprint time in CT-trained subjects. Squares represent the SMD^a^ for each trial. Diamonds represent the pooled SMD across trials.

**Fig 3:**
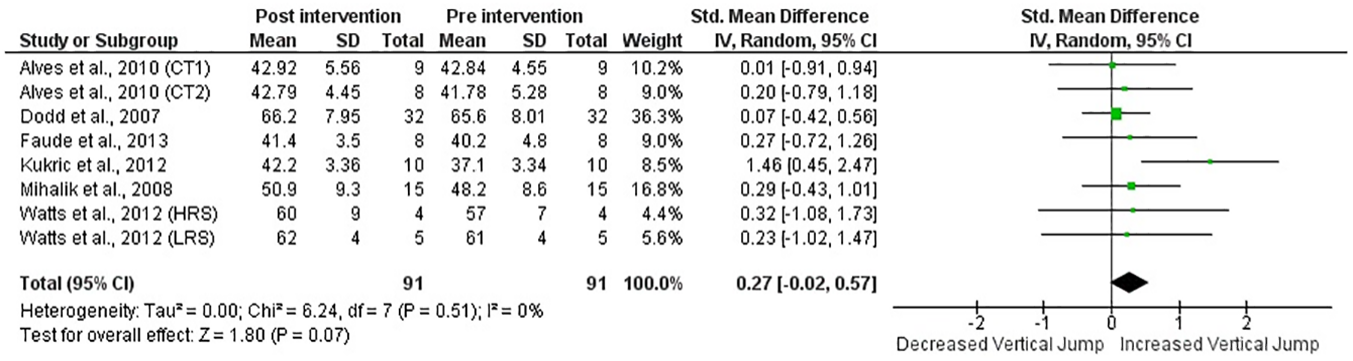
Standardized mean difference (SMD) between post and pre-intervention VJ height in CT-trained subjects. Squares represent the SMD^a^ for each trial. Diamonds represent the pooled SMD across trials.

**Fig 4:**
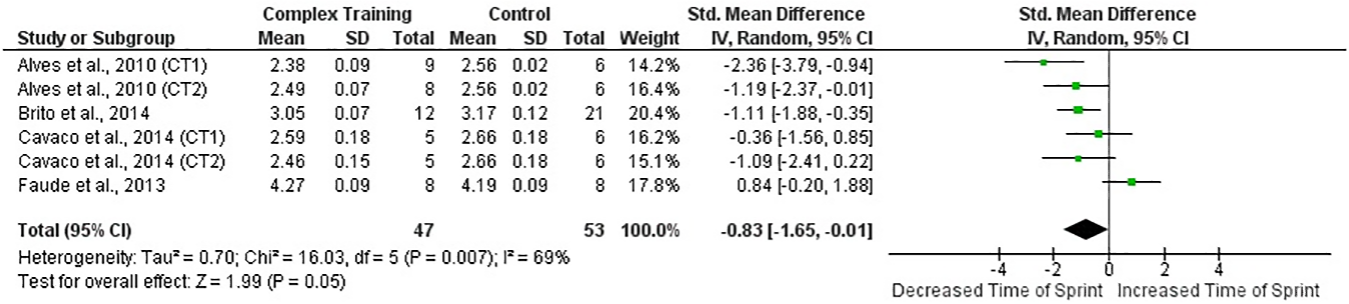
Standardized mean difference (SMD) in post-intervention sprint time between CT-trained and control subjects. Squares represent the SMD^a^ for each trial. Diamonds represent the pooled SMD across trials.

**Fig 5:**
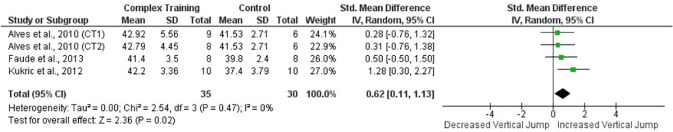
Standardized mean difference (SMD) in post-intervention VJ between CT-trained and control subjects. Squares represent the SMD^a^ for each trial. Diamonds represent the pooled SMD across trials.

When all studies and respective CT groups were examined, results indicated medium training effects (ES = 0.73) on sprint performance (p≤0.05) and small (ES = 0.34) on VJ height (p = 0.07) following CT interventions (Figs 2 and 3). Furthermore, in the studies that presented a CG, experimental groups presented better post-intervention sprint time (ES = 1.01; p = 0.05) and VJ height (ES = 0.63; p = 0.02) than CG (Figs 4 and 5).

### Subgroup analysis

Subgroup analysis assessing potential moderating factors for sprint time and VJ height are presented in Table 3. Regarding age, large ES were obtained for younger player (<20 years) in sprint (ES = 1.13) and small in VJ (ES = 0.42), independent of level of practice. For players over 20 years old, small ESs were found (sprint = 0.23; VJ = 0.20). With respect to the level of practice, an athlete was considered Division I (D1) if he was competing in first division of his respective sport, independent of the age category. All the players not competing in first division were considered under-Division I (U-D1). On sprint, both D1 (ES = 0.76) and U-D1 (ES = 0.70) athletes obtained medium training effects, independent of age On VJ, D1 athletes exhibited small ESs (0.2) and U-D1 medium (ES = 0.56).

**Table 3:** Subgroup analyses assessing potential moderating factors for sprint time and vertical jump height in studies included in the meta-analysis.

	Studies				Complex Training				
Group	Number^a^	References		SMD (95% CI)		*ES*	I^2^	*P*	*P*_*Diff*_
**Sprint**										
**Population characteristics**										
*Age*										
≥20 years	4	[33, 34, 36]		-0.24 (-0.58, 0.09)		0.23	0	0.16	<0.05	
<20 years	5	[35, 37, 41]		-1.33 (-2.02, -0.64)		1.13	43	<0.05		
*Level*										
Division 1	4	[34, 35]		-0.74 (-1.33, -0.15)		0.76	43	<0.05	<0.05	
Under Division 1	5	[33, 36, 37, 41]		-0.74 (-1.57, 0.08)		0.70	72	0.08		
**Exercise characteristics**										
*Frequency*										
≥3 week^-1^	2	[34]		-0.36 (-0.89, 0.16)		0.35	0	0.18	0.22	
<3 week^-1^	7	[33, 35, 36, 41]		-0.90 (-1.56, -0.23)		0.84	69	<0.05		
*Intensity*										
≥85% RM	3	[33, 34]		-1.07 (-1.82, -0.33)		0.25	70	<0.05	0.07	
<85% RM	6	[35, 36, 41]		-0.27 (-0.73, -0.20)		0.96	0	0.26		
*Duration*										
≥6 weeks	6	[33, 35, 37, 41]		-1.06 (-1.82, -0.31)		0.95	63	<0.05	0.07	
<6 weeks	3	[34, 36]		-0.29 (-0.65, 0.07)		0.29	0	0.12		
*Total n° Sessions*										
>12 sessions	4	[33, 34, 37]		-0.71 (-1.63, 0.20)		0.71	77	0.12	0.95	
≤12 sessions	5	[35, 36, 41]		-0.75 (-1.33, -0.17)		0.74	43	<0.05		
**Vertical Jump**										
**Population Characteristics**										
*Age*										
≥20 years	3	[33, 36, 39]		0.16 (-0.21, 0.54)		0.20	0	0.40	0.39	
<20 years	5	[35, 38, 40]		0.45 (-0.09, 1.00)		0.42	21	0.10		
*Level*										
Division 1	5	[35, 38, 39]		0.21 (-0.23, 0.64)		0.2	0	0.35	0.50	
Under Division 1	3	[33, 36, 40]		0.52 (-0.28, 1.33)		0.56	66	0.20		
**Exercise characteristics**										
*Intensity*										
≥85% RM	5	[35, 36, 38]		0.11 (-0.25, 0.48)		0.15	0	0.55	0.58	
<85% RM	3	[33, 39, 40]		0.63 (-0.10, 1.35)		0.64	49	0.09		
*Duration*										
≥6 weeks	4	[33, 35, 40]		0.47 (-0.16, 1.10)		0.45	41	0.15	0.42	
<6 weeks	4	[36, 38, 39]		0.16 (-0.21, 0.54)		0.22	0	0.39		
*Total n sessions*										
>12 sessions	2	[33, 40]		0.86 (-0.31, 2.02)		0.81	63	0.15	0.25	
≤12 sessions	6	[35, 36, 38, 39]		0.15 (-0.18, 0.47)		0.18	0	0.37		
*Intracomplex Rest*										
≥2 minutes	4	[38–40]		0.58 (-0.01, 1.18)		0.55	24	<0.05	0.21	
<2 minutes	2	[33, 36]		0.11 (-0.33, 0.55)		0.15	0	0.61		
*Sport modality*										
Jump predominance (Basketball/Volleyball)	4	[38–40]		0.58 (-0.01, 1.18)		0.55	24	0.05	0.18	
Other Team -Sports	4	[33, 35, 36]		0.11 (-0.26, 0.48)		0.12	0	0.56		

Subgroup analyses are performed on SMD between post and pre-intervention sprint time and vertical jump in CT-trained groups. Median values of continuous variables were used as cut-off values for grouping studies. Changes in moderating factors were calculated as post-intervention minus pre-intervention values.

^a^Number of CT-Trained groups into this studies references. Certain enrolled studies were not included because the value used for subgroup analysis was not reported in them.

SMD, standardized mean difference; I^2^, heterogeneity; ES, effect size; P, test for overall effect; P_Diff_, test for subgroup differences.

Concerning training frequency, from all the studies that had VJ as an outcome variable, only one [35] had a frequency other than 2 times/week. Hence, subgroup analysis was only performed for sprint. Lower training frequencies induced a large training effect on sprint performance (ES = 0.84) whereas training 3 or more times/week exhibited small ESs (ES = 0.35).

Regarding the CA intensity, large ES (ES = 0.96) on sprint time was attained for intensities below 85% 1RM and small (ES = 0.25) for higher intensities (≥85%). As for VJ, results indicated a medium ES (ES = 0.64) with loads lighter than 85% 1RM. When the workout comprised loads heavier than 85% 1RM, negligible ES were found (ES = 0.15).

Regarding the duration of intervention, longer CT programs (≥6 weeks) presented large ESs for sprint (ES = 0.95) and small for VJ (ES = 0.45) while shorter training periods (<6 weeks) showed small ESs (sprint = 0.29 and VJ = 0.22).

Regarding the number of sessions, performing less than 12 resulted in a medium training effect (ES = 0.74) for sprint and a negligible for VJ (ES = 0.18). Completing more than 12 sessions displayed a medium effect (ES = 0.71) for sprint and a large for VJ (ES = 0.81).

With reference to intracomplex rest interval (ICRI), for sprint, 2studies [35, 41] did not specify the rest between the CA and the subsequent exercise and from the remaining investigations [33, 34, 36, 37], just one presented a different rest interval [34]. Hence, no subgroup analysis was conducted for this variable. Regarding VJ, intervals longer than 2 min produced larger ESs (ES = 0.55) than shorter rest periods (ES = 0.15). However, 2 studies [35, 41] that did not report the time between the CA and the subsequent exercise were not considered in this subgroup analysis.

Finally, in relation to sport modality, athletes from team-sports in which jumping actions are more frequent and crucial for performance (basketball/volleyball) achieved medium training effects (ES = 0.55) after a CT intervention and players from other team-sports, negligible (ES = 0.12).

### Evaluation of potential bias

At evaluation of potential bias, visual interpretation of the funnel plot for the SMD between pre and post intervention sprint time and VJ height in CT participants was considered notably symmetrical, suggesting the absence of a significant publication bias. Similar results were obtained for the evaluation of potential bias of the SMD in post-intervention sprint time and VJ height between CT and CG athletes.

## Discussion

To the best of our knowledge, this is the first meta-analysis focusing on the short-term adaptations on sprint and VJ performance following CT in team-sports. The main findings indicated that this type of training lead to positive medium effects on sprint performance, over distances between 15 and 30 m. Regarding VJ height, small but positive effects were also found. Our results support the idea that CT, consisting on heavy resistance exercises coupled with plyometric/explosive exercises, set for set, on the same session, contributes to enhanced sprint and VJ performance [18–20]. The training variables that seem to most influence this positive response to CT in team-sports are the duration of intervention (≥6 weeks), the CA intensity (<85% 1RM) and the ICRI (≥2 min).

A second finding within the present meta-analysis is that, in the studies where a CG was present [33, 35, 37, 40, 41], intervention groups performed better than CG in both sprint and VJ. This is an interesting discovery given that players in CT and CG performed the same team practices, most probably containing short accelerations, sprinting, jumping and other high intensity actions characteristic of team-sports [6, 50, 51]. Therefore, we may assume that the increments found in sprint ability and VJ were due to the CT stimulus and not to the team practice [52].

An examination of the included studies shows discrepancies regarding sprint and VJ adaptations to CT. Therefore, due to such inconsistencies found in literature, the subgroup analysis performed focused on identifying potential moderating factors explaining the dissimilar adaptations following CT.

### Age and level

The present meta-analysis showed that the ESs for sprint and VJ adaptations following CT interventions were greater in younger players (<20 years), independent of the level of practice. It is possible that younger players had no sufficient strength training background, and for that reason any training stimulus would promote positive adaptations in performance, with or without PAP or combination of loads [26]. In fact, in the study by Brito et al. [37], in which CT was compared to resistance training alone and plyometric only programs, no differences were found between protocols.

Concerning level of practice, D1 players showed slightly higher ES (ES = 0.76) than U-D1 (ES = 0.70) for sprint. Previous data [53] showed that increments in sprint level of practice. However, the positive medium effects obtained by both subgroups suggest that CT may be a suitable option to increase sprint performance independent of the athletes’ level. As for VJ, U-D1 (ES = 0.42) and D1 players (ES = 0.20) presented small ESs, independent of age. It has been demonstrated that elite soccer players have higher percentages of fast muscle fibers compared to non-elite [54] and that strength levels [26, 55] and fiber type composition [56] may influence the magnitude of PAP, a possible mechanism contributing to performance gains with CT [18–20]. Also, it has been demonstrated that higher level athletes are better responders to PAP or CT programs [26, 55]. This contrasts with our findings regarding VJ, which may be possibly explained by the modest heterogeneity found in the U-D1 group, for this variable, indicating variability between the characteristics of the participants. However, reports of no differences being obtained, following CT acute protocols, among participants with dissimilar expertise, training background or strength levels have also been reported [27, 57].

### Training frequency

No analysis of training frequency was conducted for VJ since all CT groups but one (CT1 [35]) performed 2 sessions/week. On sprint, results indicated that lower training frequencies (<3 week^-1^) exhibited greater effects (ES = 0.84), than training 3 or more days. According to Seitz et al. [53], high resistance training frequencies may generate a greater stress, overwork and eventually impair performance, when performed concurrently with regular team practice. However, Seitz et al. [53] analyzed several resistance training programs and not only CT protocols. When considering solely CT, previous research [35, 41] indicated that a frequency of 2 or less times/week is as effective in increasing sprint performance as 3 or more sessions/week. Moreover, when a certain body part is actively used during competition or sport-specific training, lower weekly frequencies are needed to maintain performance levels [58].

### Duration of intervention and total number of sessions

Concerning the duration of intervention, longer interventions were found to produce greater effects on sprint and VJ performance (sprint = 0.95; VJ = 0.45). This higher magnitude of effect in sprint seems to be in line with previous findings that stated that longer resistance-based interventions (>8 weeks) resulted in improved speed development in soccer as well as rugby and American football players [2]. In basketball players, no significant correlations were identified between program duration and increments in VJ following resistance training interventions [59]. However, it is worth noting that, on their respective reviews, both Bolger et al [2] and Sperlich et al. [59] referred to various resistance-based methods and not only to CT programs. On a practical perspective, the large effect (ES = 0.95) observed on sprint performance for programs over 6 weeks seem to indicate that, adaptation wise, longer CT program should be recommended. Also, 6 weeks of duration may be a good reference for strength and conditioning professionals in terms of program duration.

With respect to the number of sessions, for sprint, 12 or less CT sessions displayed a medium training effect (ES = 0.74), as well as performing over 12 (ES = 0.71). As for VJ, the opposite was observed with a shorter number of sessions resulting in lower ESs (ES = 0.18) than interventions consisting on more than 12 workouts (ES = 0.81). However, it is important to state that only 2 CT groups performed less than 12 sessions and that a modest heterogeneity (I^2^ = 63) was found in this particular subgroup. Nevertheless, according to the data here obtained, less training sessions are needed to achieve performance improvements in sprint compared to VJ. In fact, it has been suggested that speed gains are greater when resistance training is combined with locomotor training [2]. All the participants included in the present meta-analysis were athletes currently competing in team-sports and so, apart from the CT protocols, players were engaged in sprinting actions during practice and or competition. Considering that sprinting activities are more frequent than jumping in basketball [8], rugby [5] and soccer [4, 51], it can be speculated that this is a possible rationale why, when CT is combined with regular team practice/competition, less sessions are necessary to elicit performance improvements in sprint when compared to VJ. However, further analysis of the influence of horizontally and vertically oriented exercises in CT may add valuable insight on how to maximize sprint or VJ post-intervention adaptations [60].

### Intensity of the conditioning activity

With regards to the intensity of the CA, for both variables, intensities below 85% 1RM in the CA exhibited greater training effects (sprint = 0.96; VJ = 0.64) than maximal loads (>85% 1RM; sprint = 0.25; VJ = 0.15). The type of load of the CA influences the PAP response [22, 31]. Wilson et al. [31] reported that moderate intensities, ranging from 60% to 84% 1RM produced a significantly higher PAP response than loads heavier than 85% 1RM, independent of training experience or strength levels whereas Seitz and Haff [22] indicated that maximal loads elicited greater PAP responses. It seems that PAP may be mediated by the individual’s strength level, since stronger athletes present higher PAP with maximal loads [22, 31, 55, 61, 62] while weaker subjects achieve it with sub-maximal loads [22]. It has been suggested that this occurs because when weaker individuals exercise with maximal loads, fatigue may exceed potentiation [22]. Theoretically, although PAP responses are highly individualized [20–22] and there is no clear agreement on its role as the main mechanism behind CT [24], a greater PAP could result in larger improvements on performance, following a CT protocol, if the explosive exercise was completed while the muscles were in a potentiated state [63]. With the data here obtained, it can be argued that the analyzed players’ strength levels were not high enough for them to be able to achieve greater increments on VJ performance when heavy loads were utilized, and that is why larger training effects were elicited with loads lighter that 85% 1RM.

### Intracomplex rest interval

Concerning the ICRI, a subgroup analysis was not possible to conduct for sprint performance. Two studies [35, 41] did not specify the rest between the CA and the subsequent exercise and from the remaining investigations [33, 34, 36, 37], just one presented a different rest interval [34]. For VJ, the ICRI ranged from <10 sec to 5 min. The data obtained showed that greater training effects were obtained with larger resting periods (ES = 0.55). This is in line with several studies [22, 27, 31] that have shown that the PAP response, although highly individualized, is larger when longer intervals are allowed between the CA and the subsequent explosive action. Seitz and Haff [22] indicated that rest intervals between 5 to 8 min exhibit larger PAP effects than ones ranging from 0.3 to 4 min. Nevertheless, it is worth noting that the studies reviewed by Seitz and Haff [22] were acute studies and not training interventions. This is an important aspect to consider, because when it comes to CT protocols composed by several sets of several complex pairs it is not practical to utilize ICRIs of 8 min, as the training session would take too long to be completed. Following a CA both potentiation and fatigue co-exist and the balance between these two responses is crucial if performance enhances are to be achieved [21, 22, 31]. Sale [64] identifies two dilemmas related to the PAP and fatigue responses after a CA. The first is that more intense CAs may lead to a higher potentiated state but also generate greater levels of fatigue. The second is that longer rest intervals may allow for a better recovery of fatigue but also result in a greater decrease of the PAP mechanism [64]. When it comes to designing a CT protocol it is necessary to find an adequate balance and to take into account that longer ICRIs are recommended but that in an everyday setting, recovery periods of 5–8 min may not be practical.

### Team-sport modality

The influence of sport modality was analyzed only for VJ because it was possible to differentiate among sports where jumping actions are crucial for high performance (such as basketball [6] and volleyball [65]) and other modalities (soccer, rugby or baseball). Jump predominant sports exhibited medium effects (ES = 0.55) whereas non-predominant, only small (ES = 0.12). This may be related to the specificity of training background which is known to influence performance [66], or to the fact that during training and competition, a higher number of VJ are performed by basketball [6] and volleyball [65] players in comparison to other sports [50] and that this specific stimulus lead to medium effects in the magnitude of improvement in VJ.

Regarding sprint, however, from the 9 CT intervention groups analyzed, 6 consisted on soccer players [35, 37, 41], 2 on rugby players [37] and one on baseball athletes [36]. For this reason, a subgroup analysis was not performed, as there was no modality in which sprint could be considered more crucial to performance than others.

### Limitations

Some limitations can be identified within the present meta-analysis. First, the scarce number of studies included, due to the few publications on CT interventions on team-sports that have sprint or VJ as an outcome variable. Second, not all analyzed CT programs were compared to a CG or to other training methods aimed at developing strength and/or power. Moreover, the heterogeneity in athlete characteristics (age, level, training history) is another factor that should be taken into account and that may be considered a limitation. Also, the training mechanisms outside the CT interventions were not considered in the analysis, as well as the resistance training protocols performed in the weeks prior to the CT programs. Finally, different methodological procedures and instruments were used to assess performance (VJ, particularly) in the different studies. Hence, it cannot be ruled out that some outcome values may have been affected by the method used.

## Conclusions and practical applications

CT is a training method aimed at developing both strength and power, which has a direct effect on sprint and VJ performance. When outlining the season planning for team-sports, strength and conditioning professionals should take into consideration that this may be a suitable method as it produces medium training effects on sprint performance and small positive effects on VJ.

Although the response to CT is highly individualized, based on the present results, programs lasting over 6 weeks, with a frequency of 2 sessions/week and CA activities with loads lighter than 85% 1RM seem to be the most adequate to improve sprint performance. Regarding VJ, CT protocols with a duration of more than 6 weeks, with 12 or more total sessions, CA activities below 85% 1RM and ICRI longer than 2 min appear to be the most effective on team-sport athletes. Finally, players from sports in which jumping actions are more frequent and crucial for high performance (basketball/volleyball) seem to benefit the most from CT.

## Supporting information

S1 Table PRISMA checklist. (DOC)
